# Relative contributions of arterial stiffness to cardiovascular disease risk score in Chinese women in framingham and China-PAR model

**DOI:** 10.3389/fcvm.2023.1169250

**Published:** 2023-06-15

**Authors:** Lin Jin, Jianxiong Chen, Lingheng Wu, Mengjiao Zhang, Jiali Sun, Cuiqin Shen, Lianfang Du, Dingqian Wang, Zhaojun Li

**Affiliations:** ^1^ Department of Ultrasound, Jiading Branch of Shanghai General Hospital, Shanghai Jiaotong University School of Medicine, Shanghai, China; ^2^ Department of Ultrasound, Guanghua Hospital Affiliated to Shanghai University of Traditional Chinese Medicine, Shanghai, China; ^3^ Department of Ultrasound, Mindong Hospital Affiliated to Fujian Medical University, Ningde, China; ^4^ Department of Ultrasound, Shanghai General Hospital, Shanghai Jiaotong University School of Medicine, Shanghai, China; ^5^ School of Informatics, College of Science & Engineering, The University of Edinburgh, Edinburgh, United Kingdom

**Keywords:** arterial stiffness, cardiovascular disease, risk factors, female, risk score

## Abstract

**Background:**

Arterial stiffness played an important role in the development of cardiovascular disease (CVD) events. The aim of this study was to verify the relative importance of arterial stiffness for different CVD risk scores in a large sample of Chinese women.

**Methods:**

We measured arterial velocity pulse index (AVI) and CVD risk scores in 2220 female participants (mean age 57 years). Framingham Risk Score (FRS), and the prediction for Atherosclerotic Cardiovascular Disease Risk in China (China-PAR) were used to estimate CVD risk, respectively. The relationships between AVI and risk scores were investigated by linear regressions and restricted cubic spline (RCS) analysis. To determine the relative importance of AVI in predicting CVD risk scores, random forest analysis was used.

**Results:**

There was a significant positive correlation between AVI and FRS, China-PAR in all subgroup groups stratified by age, blood pressure and BMI. AVI showed higher importance in predicting CVD risk scores in FRS model, compared with these traditional risk factors. In China-PAR model, although AVI was not as predictive as SBP, it had better predictive power than many known risk factors such as lipids. Furthermore, AVI had significant J-shaped associations both with FRS and China-PAR scores.

**Conclusions:**

AVI was significantly associated with CVD risk score. In FRS and China-PAR model, AVI showed relatively high importance in predicting CVD risk scores. These findings may support the use of arterial stiffness measurements in CVD risk assessment.

## 1. Introduction

Cardiovascular disease (CVD) poses a formidable medical challenge and stands as the leading cause of mortality among women worldwide (1). In fact, since 2000, there has been an increase in the incidence of CVD in women, including a rise in acute myocardial infarction among young women (2). Despite this, women remain underrepresented in CVD research.

CVD events share several traditional risk factors, such as hypertension, diabetes, and dyslipidemia. Moreover, arterial stiffness, recognized as an independent risk factor for CVD events, serves as a marker for vascular aging (3). Recent studies demonstrate that the arterial velocity pulse index (AVI), an assessment of arterial stiffness using cuff oscillometry, accurately reflects central artery stiffness and offers a non-invasive measure applicable in a wide array of clinical settings and large community screenings (4, 5).

The CVD risk score is the most commonly used clinical metric to evaluate patient CVD risk and determine optimal primary prevention (6). The widely accepted Framingham risk score (FRS) serves as a valuable tool for predicting ischemic cardiac disease, peripheral vascular disease, cerebrovascular disease, and risk stratification in the general population through a straightforward evaluation of demographic, clinical, and biochemical parameters (7). Nevertheless, the FRS model originated from a US study cohort and predominantly applies to US white individuals (8), potentially overestimating disease risk in European and Asian populations (9–11). Moreover, in populations with low FRS (<10%), the FRS has limited predictive value for forecasting CVD incidence, particularly in young adults and women (12).

The China-PAR project is developed to predict the 10-year risk of cardiovascular disease (CVD) in Chinese individuals, and it is currently the recommended risk assessment model in the Chinese Guidelines for Cardiovascular Risk Assessment and Management (6).

However, the relative significance of AVI across different CVD risk score models for assessing CVD risk in women remains unexplored. Thus, our aim was to analyze a large sample of Chinese women and compare the differences in using two risk scoring models (FRS and China-PAR) and assess the relative importance of AVI.

## 2. Methods

### 2.1. Study population

**Figure 1:**
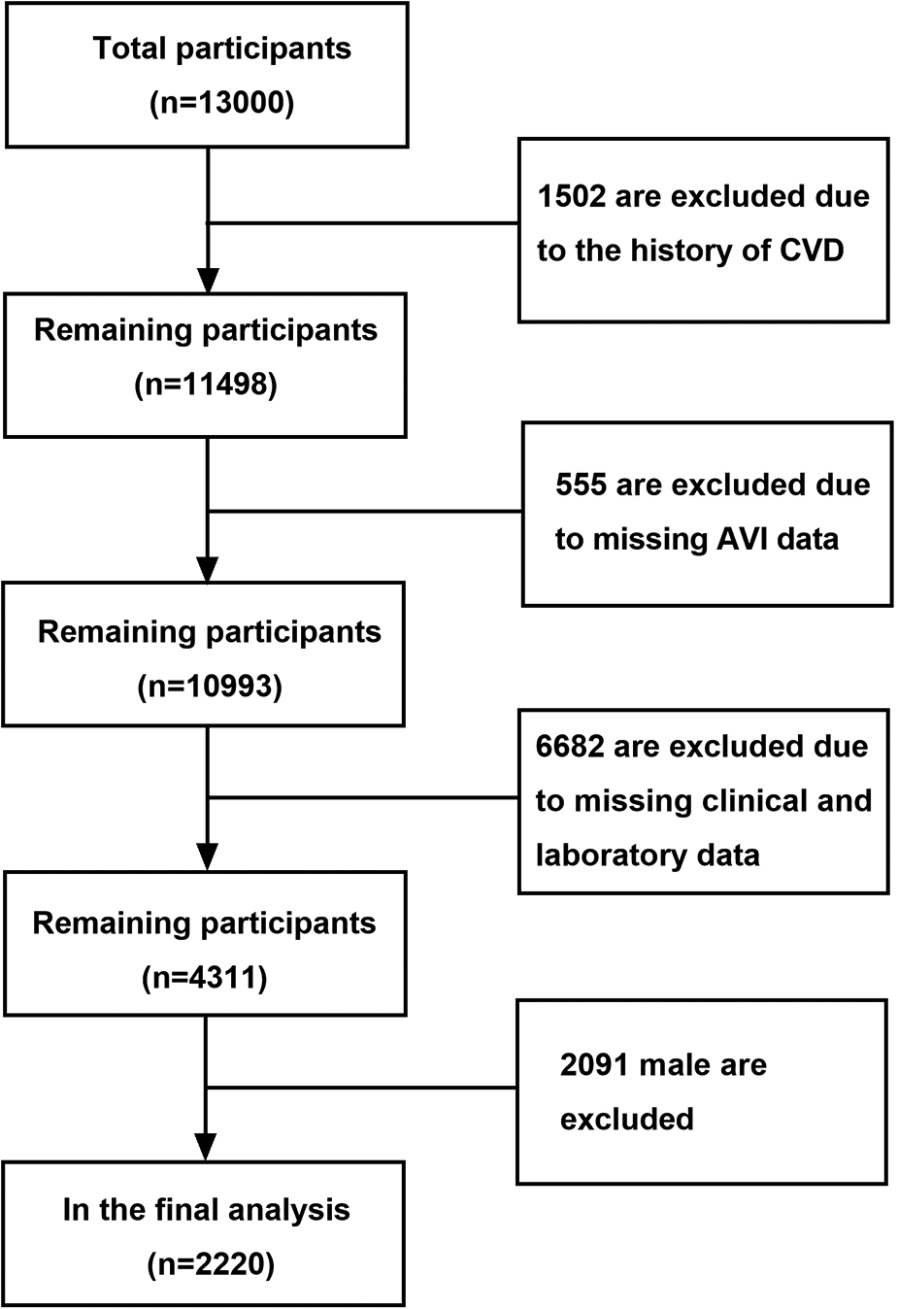
Patient selection and study design. Flow chart showing inclusion and exclusion criteria for study population. CVD, cardiovascular disease; AVI, arterial velocity pulse index.

This study was a part of a cohort study that involved Chinese men and women aged 18 years or older, who underwent a health examination at the Health Management Center in the Shanghai General Hospital Jiading Branch, China, as previously described (13). A total of 10,779 participants were initially enrolled, but those who met any of the following criteria at baseline were excluded: missing data on cuff oscillation wave parameters (*n* = 505), missing data on clinical and laboratory parameters (*n* = 6,682), a history of cardiovascular disease events (CVD, *n* = 1,502), and male gender (*n* = 2,091). Ultimately, 2,220 female individuals were included in the study (Figure 1). The study was approved by the Shanghai General Hospital Ethics Committee (No. 2021KY057) and registered on the official website of the China Clinical Trial Registration Center (ChiCTR2000035937).

### 2.2. Data collection

Data on lifestyle factors, medical and medication history, and the history of CVD were collected through standardized, self-administered questionnaires. Anthropometric parameters, blood pressure, and blood sampling were measured by trained nurses or technicians. Current smoking was defined as smoking within the past 12 months. Body mass index (BMI) was calculated as weight (kg) divided by square height (m^2^), and a BMI of ≥28 kg/m^2^ was considered as obese (14). Relevant blood test results were also obtained, including total cholesterol (TC), triglyceride (TG), low-density lipoprotein (LDL-C), high-density lipoprotein (HDL-C), and fasting blood glucose (FBG).

Hypertension was defined as systolic blood pressure (SBP) of ≥140 mmHg and/or diastolic blood pressure (DBP) of ≥90 mmHg, and/or antihypertensive medication (15). Diabetes was defined as current use of insulin or oral hypoglycemic agents, and/or glycosylated hemoglobin (Hb) of ≥6.5%, and/or FBG of ≥7.0 mmol/l (16). Dyslipidemia was defined as TC of ≥6.61 mmol/l and/or TG of ≥1.7 mmol/l after an overnight fast and/or the presence of lipid-lowering therapy (17).

### 2.3. Ten-year risk score and stratification of developing CVD

In this study, we utilized two different algorithms, namely China-PAR and FRS, to assess the 10-year risk of developing CVD. The FRS score was calculated using the NCEP-ATP algorithm (18) and incorporated variables such as gender, age, TC, smoking status, HDL-C, and SBP. This tool was applicable to individuals over the age of 30 with no history of cardio-cerebrovascular diseases. The China-PAR model, in addition to traditional risk factors (gender, age, treated or untreated systolic blood pressure, TC, HDL-C, smoking, and diabetes), also included four factors specific to China's national conditions, namely waist circumference, geographical region (southern or northern), administrative region (urban or rural), and family history of CVD (6).

Based on the predicted CVD risk score, the China-PAR model categorized CVD risk as “low” (<5%), “medium” (5%–10%), and “high” (≥10%), while the FRS model categorized CVD risk as “low” (<15%), “moderate” (15%–20%), and “high” (≥20%).

### 2.4. Arterial stiffness

To assess arterial stiffness, we used the non-invasive brachial oscillographic blood pressure cuff PASESA AVE-2000Pro (Shisei Datum, Tokyo, Japan) and measured the AVI (19). Participants were instructed to refrain from smoking, alcohol, and caffeine-containing beverages on the day of the AVI measurement, and to rest for at least 5 min before the examination. AVI was calculated by collecting cuff oscillation wave under the condition of high cuff pressure (higher than brachial artery systolic pressure). The waveform had double peaks in systole, and AVI was defined as AVI = 20 ×|V2|/|P1|. P1 was measured as the first peak of the differentiated waveform between pulse wave and time, while V2 was the absolute value of the bottom of the differentiated waveform between pulse wave and time. AVI is a dimensionless index, and we used the mean of three measurements, with at least 2 min between each measurement, for the analysis in this study.

### 2.5. Statistical analysis

Continuous variables were presented as mean ± standard deviation, categorical variables were presented as numbers (percentages). The Chi-squared test was used to analyze comparisons of categorical variables, and analysis of variance was used for continuous variables.

The correlation coefficient between CVD risk factors and FRS, China-PAR was assessed using the Spearman correlation coefficient. Multivariable linear regression analysis was performed to investigate the association between CVD risk factors and AVI, FRS and China-PAR risk score, respectively. The models adjusted for age, BMI, SBP, DBP, pulse, TG, TC, HDL-C, LDL-C and AVI. To determine the relative importance of CVD risk factors in predicting FRS and China-PAR risk score, we performed random forest analysis. The classification error for the random forest trees and the error after permuting the predictor variables were used to analyze the relative importance of each variable (20).

To detect possible non-linear relationships between AVI and FRS and China-PAR risk score, restrictive cubic spline (RCS) analyses were used, with using 5 knots according to the percentiles of the AVI distribution, as the RCS model is a smoothly joined sum of polynomial functions that do not assume linearity of the relationship (21). A *p*-value less than 0.05 was considered statistically significant. We constructed the nomogram using R software version 3.5.1 (R foundation for statistical computing, Vienna, Austria. URL http://www.R-project.org) and conducted internal verification.

## 3. Results

### 3.1. Baseline characteristics as well as AVI findings

Table 1 displays the baseline clinical characteristics of the study participants. The mean age of the subjects was 57.0 ± 12.6 years. The prevalence of hypertension, diabetes mellitus, dyslipidemia, and current smoking was 29.1%, 19.8%, 27.0%, and 1.3%, respectively. The results of AVI are also presented in Table 1, showing that the mean value of AVI in the total population was 17.96 ± 6.58.

**Table 1:** Basic characteristics of the study population (mean ± SD).

Characteristic	≤29 years	30–39 years	40–49 years	50–59 years	60–69 years	≥70 years	Total	*p* value
*n*	70	194	301	557	768	330	2,220	
Age (years)	26.01 ± 2.44	35.16 ± 2.85	44.96 ± 2.86	54.96 ± 3.00	64.46 ± 2.64	73.17 ± 2.61	56.96 ± 12.59	<0.001
**Traditional risk factors**								
Diabetes (%)	5 (7.1%)	5 (2.6%)	24 (8.0%)	97 (17.4%)	209 (27.2%)	99 (30.0%)	439 (19.8%)	<0.001
Hypertension (%)	4 (5.7%)	8 (4.1%)	20 (6.6%)	148 (26.6%)	313 (40.8%)	154 (46.7%)	647 (29.1%)	<0.001
Dyslipidaemia (%)	9 (12.9%)	21 (10.8%)	55 (18.3%)	201 (36.1%)	235 (30.6%)	79 (23.9%)	618 (27.8%)	<0.001
BMI (kg/m^2^)	22.75 ± 4.40	23.60 ± 4.45	23.89 ± 2.93	24.40 ± 3.47	24.08 ± 3.56	24.11 ± 3.37	24.06 ± 3.56	0.002
**Blood pressure, mm Hg**								
Systolic	112.21 ± 16.51	114.56 ± 18.08	120.13 ± 17.99	129.76 ± 20.65	133.67 ± 22.52	142.77 ± 26.61	129.86 ± 23.25	<0.001
Diastolic	73.37 ± 11.07	74.24 ± 11.98	76.10 ± 10.93	79.50 ± 12.05	77.43 ± 11.89	76.05 ± 13.54	77.16 ± 12.16	<0.001
Pulse (beats/min)	91.20 ± 15.54	84.73 ± 11.60	79.73 ± 11.99	79.20 ± 12.29	78.23 ± 11.65	78.47 ± 11.99	79.69 ± 12.34	<0.001
**Laboratory parameters**								
TC (mmol/L)	3.94 ± 0.85	4.13 ± 0.88	4.49 ± 0.87	4.87 ± 1.01	4.74 ± 1.01	4.41 ± 1.06	4.61 ± 1.02	<0.001
TG (mmol/L)	0.95 ± 0.56	1.06 ± 0.67	1.39 ± 1.04	1.52 ± 1.08	1.44 ± 0.77	1.59 ± 1.03	1.42 ± 0.94	<0.001
HDL cholesterol (mmol/L)	1.18 ± 0.30	1.19 ± 0.28	1.22 ± 0.32	1.23 ± 0.30	1.25 ± 0.34	1.14 ± .29,947	1.22 ± 0.32	<0.001
LDL cholesterol (mmol/L)	2.40 ± 0.86	2.59 ± 0.81	2.79 ± 0.82	3.10 ± 0.93	2.92 ± 0.95	2.65 ± 1.00	2.86 ± 0.94	<0.001
Fasting blood glucose (mmol/L)	4.87 ± 0.89	5.04 ± 0.71	5.29 ± 1.39	5.71 ± 1.53	5.83 ± 1.57	5.99 ± 1.79	5.65 ± 1.53	<0.001
**Arterial stiffness parameters**								
AVI	11.26 ± 3.30	12.18 ± 3.89	15.66 ± 5.80	18.35 ± 6.09	19.37 ± 6.34	20.97 ± 6.78	17.96 ± 6.58	<0.001
**10-year CVD risk score**								
Framingham Risk Score	/	−1.00 (−4.25–2.00)	6.00 (4.00–8.00)	12.00 (10.00–14.00)	15.00 (13.00–16.00)	19.00 (17.00–20.00)	13.00 (9.00–16.00)	<0.001
China-PAR	0.05 (0.02–0.12)	0.34 (0.11–0.59)	1.40 (0.79–2.63)	5.06 (3.19–7.93)	10.14 (6.76–14.34)	18.29 (13.38–24.47)	6.44 (1.69–12.50)	<0.001

Data are given as means ± SD. SD, standard deviation.

### 3.2. CVD risk factors and ten-year risk score

**Figure 2:**
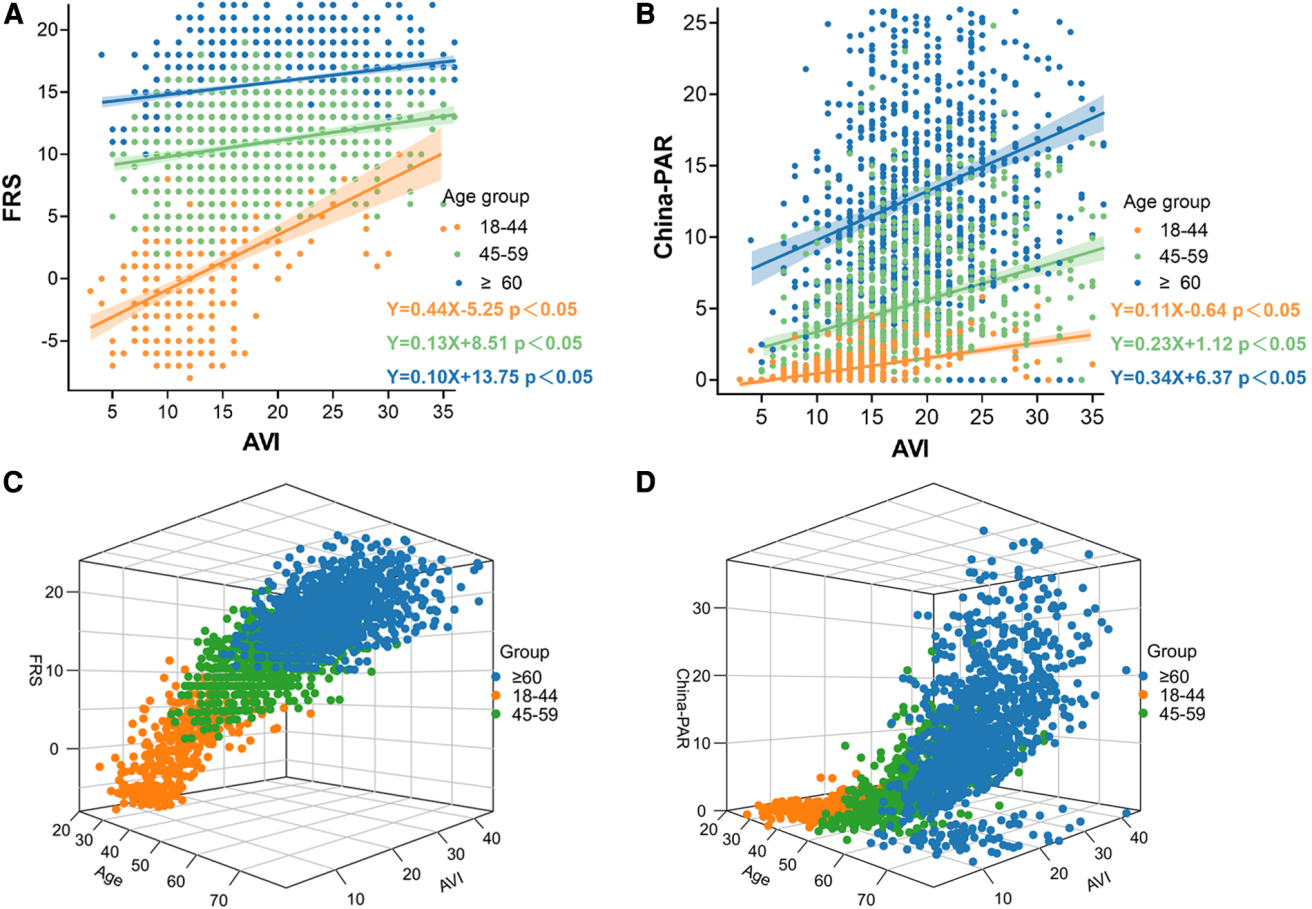
Correlation between AVI and FRS and China-PAR in groups by age. (**A**) Associations between Framingham Risk score and AVI by age groups; (**B**) Associations between China-PAR score and AVI by age groups; (**C**) Age/AVI relations of Framingham Risk score, age (x axis, years), and AVI (y axis) and FRS; Age, AVI and interaction with FRS are highly significant; (**D**) Age/AVI relations of China-PAR score, age (x axis, years), and AVI (y axis) and China-PAR; Age, AVI and interaction with China-PAR are highly significant.

**Figure 3:**
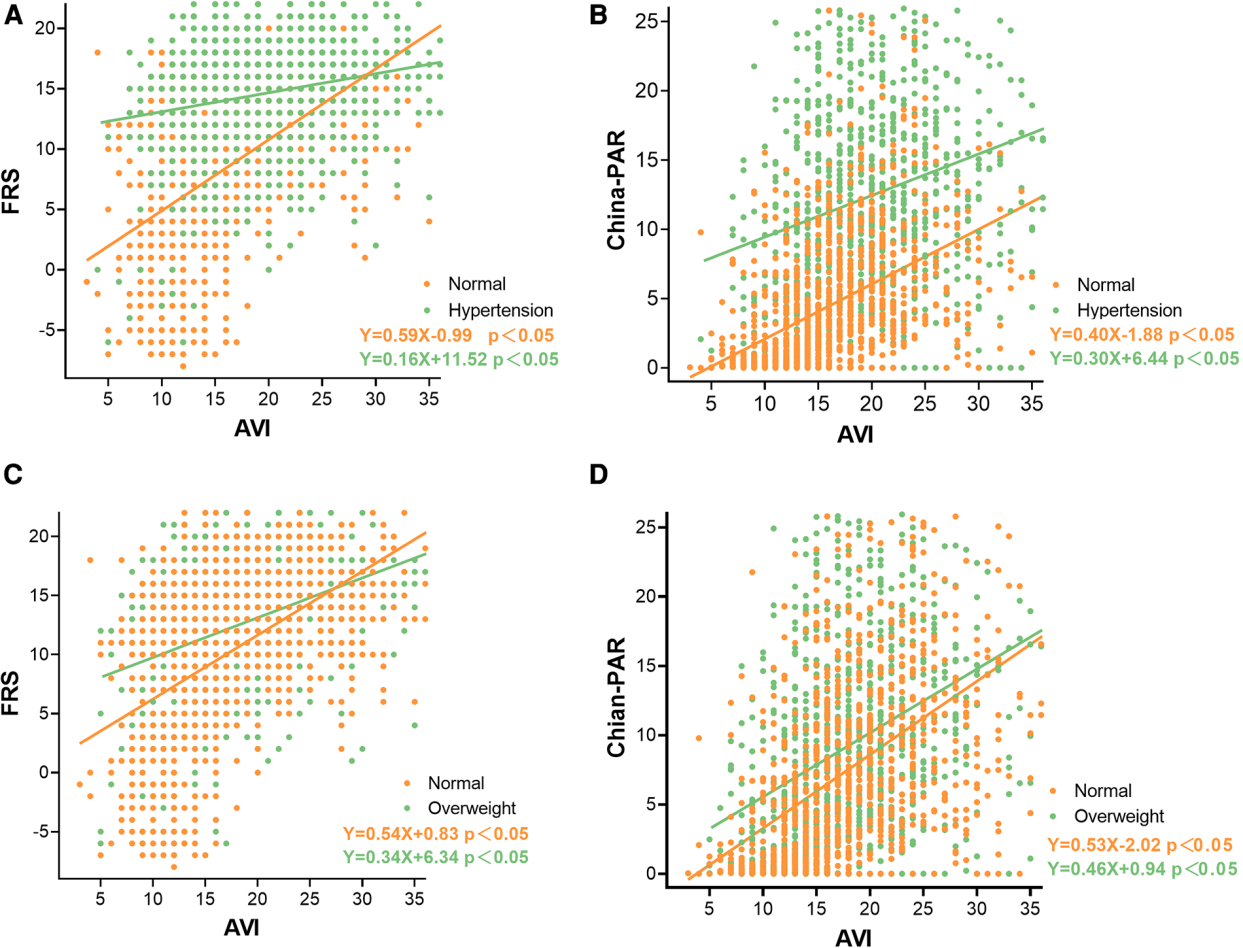
Correlation between AVI and FRS and China-PAR in groups by blood pressure and BMI. (**A**) Associations between Framingham Risk score and AVI by blood pressure groups; (**B**) Associations between China-PAR score and AVI by blood pressure groups; (**C**) Associations between Framingham Risk score and AVI by BMI groups; (**D**) Associations between China-PAR score and AVI by BMI groups.

The distribution of clinical characteristics based on different risk stratification and in patients younger than 50 years were compared in Supplementary Tables S1, S2. There was a significant positive correlation between AVI and FRS, China-PAR in all subgroup groups stratified by age, blood pressure and BMI. The correlations in the age groups of young (18–44 years old), middle-aged (45–59 years old), and old (≥60 years old) were shown in Figure 2. The correlations in the blood pressure and BMI subgroups were shown in Figure 3.

### 3.3. Association between CVD risk factors and AVI and ten-year risk score

We further assessed the association between CVD risk factors and AVI in linear regression models, and the beta-coefficients and 95% CI of both adjusted models were shown in Table 2. Stepwise multivariate regression analysis showed that age, SBP, BMI, pulse and HDL-C were independent factors associated with AVI, although TC, TG, LDL-C and FBG were not associated with AVI.

**Table 2:** Association between CVD risk factors and AVI in multivariate linear analyses.

		AVI	
Items	*β*	95% CI	*p* value
Age	0.215	0.093, 0.132	<0.001
SBP (mmHg)	0.415	0.107, 0.128	<0.001
Pulse (beats/min)	−0.230	−0.141, −0.104	<0.001
BMI (kg/m^2^)	−0.070	−0.195, −0.064	<0.001
HDL cholesterol (mmol/l)	0.040	0.110, 1.538	0.024

β is the regression coefficient.

We investigated the associations between AVI and FRS, China-PAR in the linear regression analysis to determine the presence of a confounding effect by age, SBP, BMI, pulse and HDL-C (Tables 3, 4). AVI was not associated with China-PAR after adjustment for risk factors. However, this does not fully explain the discrepancy.

**Table 3:** Univariate and multivariate analysis to the association between FRS and other variables.

Variable	Univariate		Multivariable	
	r	*p* value	β(95% CI)	*p* value
Age	0.910	<0.001	0.433 (0.425,0.441)	<0.001
BMI	0.132	<0.001	0.102 (0.077,0.128)	<0.001
SBP (mmHg)	0.538	<0.001	0.055 (0.050,0.059)	<0.001
Pulse	−0.090	<0.001	0.005(−0.003,0.012)	0.209
AVI	0.434	<0.001	0.028 (0.012,0.044)	0.001
HDL-C(mmol/l)	−0.081	<0.001	−0.814(−1.089,−0.540)	<0.001

**Table 4:** Univariate and multivariate analysis to the association between China-PAR and other variables.

Variable	Univariate		Multivariable	
	r	*p* value	β(95% CI)	*p* value
Age	0.762	<0.001	0.333 (.316,0.350)	<0.001
BMI (kg/m^2^)	0.117	<0.001	−0.088(−0.142,−0.034)	0.001
SBP (mmHg)	0.737	<0.001	0.164 (0.154,0.174)	<0.001
Pulse	−0.038	0.087	0.001(−0.015,0.017)	0.899
AVI	0.425	<0.001	−0.021(−0.055,0.013)	0.232
HDL-C(mmol/l)	−0.160	<0.001	−2.953(−3.541,−2.365)	<0.001

β is the regression coefficient.

### 3.4. Agreement between two risk categories

The China-PAR algorithm classified around one-third of participants as high-risk (35.6%), and the FRS classified less than one-tenth as high-risk (5.1%).

Due to the overlap of CVD risk factors, there was a significant overlap between the FRS and China-PAR risk scores (Kappa 0.356, *p* < 0.001), as shown in Table 5, and this alignment was still observed in under-50 and over-50 age groups (Supplementary Table S3). Although the similarity/overlap is significant and consistent, there is also non-negligible discrepancy between FRS and China-PAR, due to the additional three factors added in China-PAR for Chinese population.

**Table 5:** Agreement between the two risk category.

China-PAR risk category	Framingham risk category				
	Low risk	Medium risk	High risk	Kappa	*p* value
Low risk	609 (27.4%)	345 (15.5%)	0 (0.0%)	0.356	<0.001
Medium risk	9 (0.4%)	473 (21.3%)	0 (0.0%)		
High risk	0 (0.0%)	671 (30.2%)	113 (5.1%)		

### 3.5. Relative importance of AVI on CVD risk score

**Figure 4:**
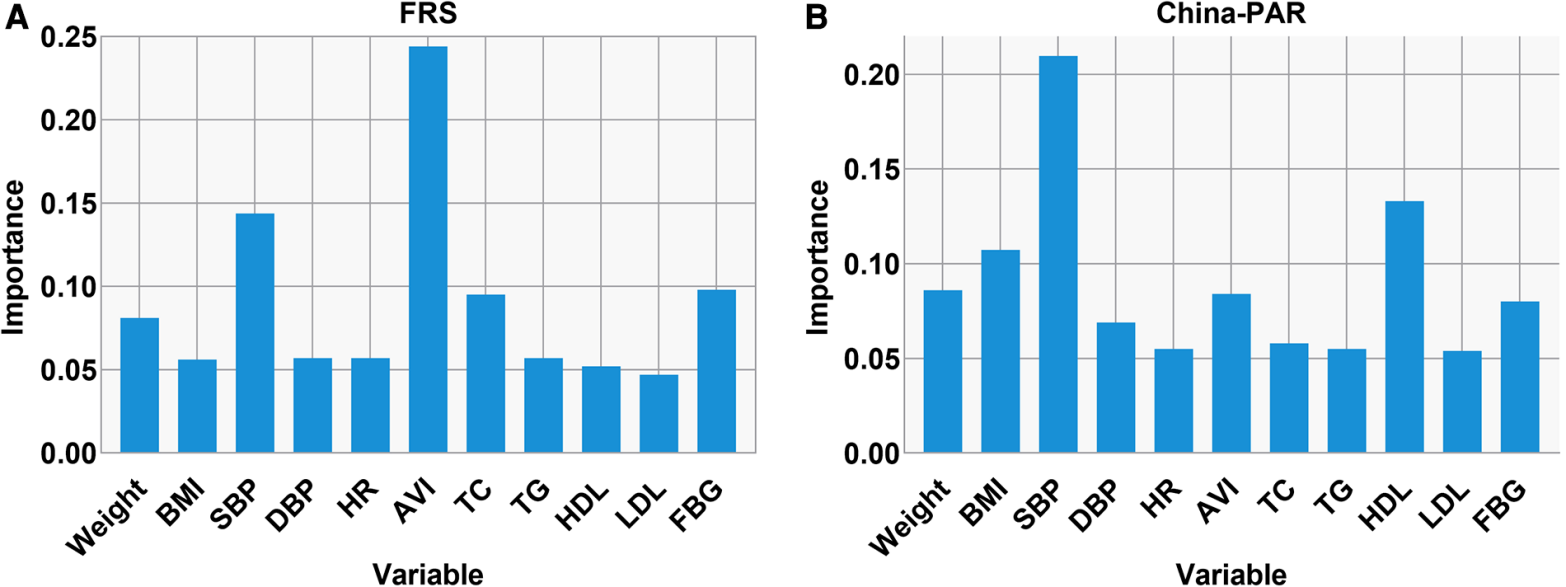
Relative importance of AVI in predicting the CVD risk score. Relative importance of AVI in predicting the high FRS (**A**) and China-PAR (**B**) as analyzed in random forest analysis. The top 11 important variables are depicted.

In order to assess the relative importance of AVI in relation to traditional cardiovascular risk factors, we conducted a random forest analysis. AVI showed higher importance in FRS model, compared with these traditional risk factors, which indicated the high predictive power of AVI. In China-PAR model, although AVI was not as predictive as SBP, it had better predictive power than many known risk factors such as LDL-C, TC and TG. These findings are presented in Figures 4A,B, respectively.

### 3.6. Factors associated with CVD risk among under 30 years old female

Considering the inclusion criteria of the Framingham algorithm, which required participants to be aged 30 years or older, our study observed a specific percentage of women under 30 years of age who exhibited risk factors linked to CVD, such as dyslipidemia, obesity (7.1%), and diabetes (7.1%) (Supplementary Table S4).

### 3.7. Association between AVI and FRS and China-PAR

**Figure 5:**
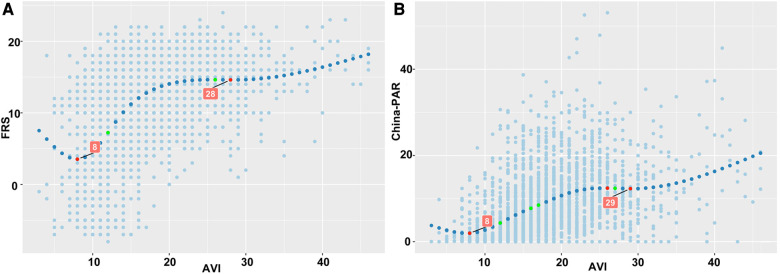
Relationships between AVI and FRS and China-PAR score. (**A**) There was a significant J-shaped relationship between AVI and FRS scores, with FRS scores increasing from AVI 8 units and then increasing rapidly again when AVI reached 28 units. (**B**) There was a significant J-shaped relationship between AVI and China-PAR scores, with China-PAR scores increasing from AVI 8 units and then increasing rapidly when AVI reached 29 units.

The RCS analysis curves of AVI and FRS and China-PAR showed that AVI had significant J-shaped associations with both FRS and China-PAR scores. The FRS and China-PAR scores started to increase from AVI 8 units, and when AVI reached 29 units for China-PAR and 28 units for FRS, they increased rapidly again, as shown in Figure 5.

## 4. Discussion

This current cohort study evaluated the association between AVI and CVD risk scores in Chinese women based on FRS and China-PAR scoring models. A Our cohort study revealed a significant association between AVI and both FRS and China-PAR scores. Interestingly, we observed a significant J-shaped association between AVI and both scoring models. Notably, in the FRS model, AVI became a more prominent risk factor. In addition, we established the agreement between two risk categories. Furthermore, we assessed the agreement between the two risk categories and found that although the similarity/overlap was significant and consistent, there was also non-negligible discrepancy between FRS and China-PAR. These findings provided important insights into the role of AVI in CVD risk assessment.

Our study showed a significant J-shaped association between AVI and both FRS and China-PAR scores, with consistent inflection points for both models. Our previous study suggested that this J-shaped relationship may be related to age and the development of vascular function in early life (4, 22). As we age, the large elastic vessels in our bodies undergo progressive luminal dilatation, thickening of the arterial wall, and increased deposition of collagen. Additionally, the fragmentation and degeneration of elastin further reduce the vessels' capacity to dampen blood pulsatility caused by the heart's contractions. These changes ultimately lead to an increase in arterial stiffness and SBP (23).

Previous studies showed that AVI was associated with known risk factors for CVD (24, 25). In the current study, AVI was independently associated with age, SBP, BMI, pulse, and HDL-C. AVI, which was derived from the amplitude of oscillometric reflected waveforms and reflected stiffness of the central arteries (26) was influenced by age and peripheral arterial resistance. Thus, AVI was strongly correlated with age and SBP.

In addition, we further analyzed the relationship between AVI and CVD risk scores stratified by CVD risk factors. In this analysis, the results were consistent. AVI was significantly associated with FRS and China-PAR in all subgroup groups stratified by age, blood pressure and BMI.

Interestingly, we observed that we observed a higher correlation between AVI and FRS in younger patients than in China-PAR, and the reverse in older patients. Another interesting finding of our study was that AVI was a more significant factor affecting FRS among CVD risk factors, while SBP was more important for China-PAR. The awareness, treatment, and control of hypertension have been improving in the general US adult population since the 1960s (27, 28). The Framingham Heart Study showed that a proportion of treated hypertensives still had high arterial stiffness, and well-controlled hypertension still had a notable residual CVD risk (29). However, in China, hypertension remains a significant public health concern, with a large proportion of the population having inadequate control of the condition (30). Although hypertension prevalence has declined slightly since 2010, there are still significant disparities in hypertension awareness, treatment, and control across different regions and socioeconomic groups (31, 32). In addition, numerous studies showed that many of the traditionally modifiable risk factors, such as lipids, can often be normalized by aggressive drug therapies that also improve CVD risk (33). While, stiffness was usually considered as an index of vascular aging (34), may be irreversible (29). Therefore, prioritizing primary prevention strategies is crucial in managing hypertension in China. However, in China-PAR, AVI had better predictive power than many known risk factors such as LDL, TC and TG.

Since different CVD risk assessment models were derived from different cohorts, they may include different risk factors and endpoint events, resulting in varying scopes of application. Therefore, in the future, using AVI as an indicator of arterial stiffness in CVD risk assessment may provide additional information for CVD assessment, and may identify more asymptomatic women who may benefit from more aggressive primary preventive treatment.

## 5. Strengths and limitations

Our study has several strengths, including the use of various statistical techniques such as RCS and random forest analysis to demonstrate the association between arterial stiffness and CVD risk scores in a large cohort of female participants. However, there are also some limitations that must be considered. Firstly, since this was a single-center study, further multicenter prospective studies are needed to validate the random forest analysis model in the future. Secondly, AVI was a new oscillometric indices of arterial stiffness and reflected the central arterial stiffness (35, 36). However, AVI cannot reflect all arterial stiffness in the body. Further investigation is needed to clarify the clinical value of AVI. Thirdly, the present study mainly considered the aging-related impact, while considering the hormonal changed from premenopausal to postmenopausal in females, and the antihypertensive drugs and statin may play a role in the arterial stiffening progression, and our further studies should include different subgroups to analyze the substantial impact of these factors on arterial aging. Furthermore, we did not include male patients in the present study, although recent studies showed sex-specific was a determinant of artery stiffness independent of age and blood pressure, and females were more vulnerable to the progression of arterial aging (37).

## 6. Conclusions

AVI was significantly associated with CVD risk score. In FRS and China-PAR model, AVI showed relatively high importance in predicting CVD risk scores, and had significant J-shaped associations with FRS and China-PAR scores. These findings may support the use of arterial stiffness measurements in CVD risk assessment.

## Data Availability

The original contributions presented in the study are included in the article/**Supplementary Material**, further inquiries can be directed to the corresponding author.
