# Supplementary material for: Relative contributions of arterial stiffness to cardiovascular disease risk score in Chinese women in framingham and China-PAR model
Source: Front Cardiovasc Med. 2023 Jun 15;10:1169250. doi: 10.3389/fcvm.2023.1169250 (PMC10311511; doi:10.3389/fcvm.2023.1169250)
Supplement: Supplementary file 1 [file Table1.docx]

| **Supplementary Table 1. Characteristics according to the two-risk score model** | | | | | | | | |
| --- | --- | --- | --- | --- | --- | --- | --- | --- |
| **Item** | **Framingham** | | | | **China-PAR** | | | |
|  | **Low risk** | **Medium risk** | **High risk** | **p value** | **Low risk** | **Medium risk** | **High risk** | **p value** |
| n | 548 | 1489 | 113 |  | 880 | 480 | 790 |  |
| Age (years) | 42.71±6.83 | 62.33±6.82^*^ | 74.43±2.89^*#^ | ＜0.001 | 48.30±9.77 | 60.21±5.64^*^ | 67.36±6.04^*#^ | ＜0.001 |
| **Traditional risk factors** |  |  |  |  |  |  |  |  |
| Diabetes (%) | 39 (7.1%) | 354 (23.8%) | 41 (36.3%) | ＜0.001 | 80 (9.1%) | 89 (18.5%) | 265 (33.5%) | ＜0.001 |
| Hypertension (%) | 39 (7.1%) | 537 (36.1%) | 67 (59.3%) | ＜0.001 | 109 (12.4%) | 155 (32.3%) | 379 (48.0%) | ＜0.001 |
| Dyslipidaemia (%) | 51 (9.3%) | 510 (34.3%) | 30 (26.5%) | ＜0.001 | 193 (21.9%) | 162 (33.8%) | 236 (29.9%) | ＜0.001 |
| **Anthropometrics** |  |  |  |  |  |  |  |  |
| Weight (Kg) | 60.47±9.80 | 60.94±9.87 | 60.26±8.54 | 0.270 | 60.42±9.97 | 60.94±9.78 | 61.10±9.58 | 0.174 |
| BMI (kg/m^2^) | 23.64±3.53 | 24.22±3.53^*^ | 24.74±3.16^*^ | ＜0.001 | 23.71±3.52 | 24.19±3.75^*^ | 24.47±3.34^*^ | 0.001 |
| Systolic blood pressure (mm Hg) | 115.92±16.30 | 133.15±21.52^*^ | 165.02±23.61^*#^ | ＜0.001 | 116.17±17.70 | 127.36±15.20^*^ | 148.20±20.74^*#^ | ＜0.001 |
| Diastolic blood pressure (mm Hg) | 74.20±10.68 | 78.11±12.20^*^ | 81.21±15.41^*^ | ＜0.001 | 74.08±11.05 | 77.69±11.47^*^ | 80.60±12.84^*#^ | ＜0.001 |
| HR (beats/min) | 81.27±11.93 | 78.63±11.98^*^ | 78.89±12.34^*^ | ＜0.001 | 80.08±11.76 | 78.35±12.02^*^ | 79.05±12.32^*^ | ＜0.001 |
| **Laboratory parameters** |  |  |  |  |  |  |  |  |
| TC (mmol/L) | 4.21±0.78 | 4.80±1.04^*^ | 4.59±1.09^*^ | ＜0.001 | 4.52±0.95 | 4.83±1.03^*^ | 4.66±1.06^*#^ | ＜0.001 |
| TG (mmol/L) | 1.18±0.79 | 1.50±0.95^*#^ | 1.91±1.19^*#^ | ＜0.001 | 1.25±0.76 | 1.50±1.00^*^ | 1.61±1.05^*^ | ＜0.001 |
| HDL cholesterol (mmol/L) | 1.22±0.31 | 1.23±0.32 | 1.03±0.23^*#^ | ＜0.001 | 1.24±0.33 | 1.26±0.30 | 1.17±0.31^*#^ | ＜0.001 |
| LDL cholesterol (mmol/L) | 2.57±0.75 | 2.99±0.97^*^ | 2.83±1.01^*^ | ＜0.001 | 2.80±0.86 | 3.00±0.98^*^ | 2.88±1.00^*^ | ＜0.001 |
| FBG (mmol/L) | 5.23±1.20 | 5.80±1.58^*^ | 6.27±1.93^*#^ | ＜0.001 | 5.33±1.25 | 5.68±1.36^*^ | 6.06±1.82^*#^ | ＜0.001 |
| **Arterial stiffness parameters** |  |  |  |  |  |  |  |  |
| AVI | 14.41±5.21 | 19.22±6.34^*^ | 22.82±7.16^*#^ | ＜0.001 | 15.17±5.37 | 18.77±5.84^*^ | 21.18±6.67^*#^ | ＜0.001 |

Compared with the low risk group, ^*^ p＜0.05; Compared with the medium risk group, ^#^ p＜0.05

**Supplementary Table 2. Characteristics for the two-risk score model** **in patients younger than 50 years**

| **Characteristic** | **Framingham** | | | **China-PAR** | | | |
| --- | --- | --- | --- | --- | --- | --- | --- |
|  | **Low risk** | **Medium risk** | **p value** | **Low risk** | **Medium risk** | **High risk** | **p value** |
| n | 522 | 43 |  | 549 | 12 | 4 |  |
| Age (years) | 38.66±7.20 | 46.42±2.76 | ＜0.001 | 39.05±7.24 | 45.83±4.22^*^ | 47.75±1.50^*^ | ＜0.001 |
| **Traditional risk factors** |  |  |  |  |  |  |  |
| Diabetes (%) | 27(5.2%) | 7(16.3%) | 0.010 | 27(4.9%) | 6(50.0%) | 1(25.0%) | ＜0.001 |
| Hypertension (%) | 22(4.2%) | 10(23.3%) | ＜0.001 | 26(4.7%) | 3(25.0%) | 3(75.0%) | ＜0.001 |
| Dyslipidaemia (%) | 54(10.3%) | 31(72.1%) | ＜0.001 | 76(13.8%) | 6(50.0%) | 3(75.0%) | ＜0.001 |
| **Anthropometrics** |  |  |  |  |  |  |  |
| BMI (kg/m^2^) | 23.50±3.71 | 25.52±3.39 | 0.001 | 23.57±3.71 | 26.15±3.28^*^ | 27.29±2.86^*#^ | 0.008 |
| Systolic blood pressure (mm Hg) | 115.69±16.75 | 136.05±22.89 | ＜0.001 | 115.96±16.53 | 155.50±12.40^*^ | 177.75±7.97^*#^ | ＜0.001 |
| Diastolic blood pressure (mm Hg) | 74.33±10.88 | 84.79±12.62 | ＜0.001 | 74.58±11.02 | 93.00±4.99^*^ | 96.25±5.85^*^ | ＜0.001 |
| HR (beats/min) | 82.85±12.75 | 83.09±15.10 | 0.905 | 82.85±12.73 | 85.67±19.18 | 77.25±20.02 | 0.518 |
| **Laboratory parameters** |  |  |  |  |  |  |  |
| TC (mmol/L) | 4.19±0.81 | 5.63±0.84 | ＜0.001 | 4.28±0.89 | 4.99±1.08^*^ | 5.32±0.36^*#^ | 0.002 |
| TG (mmol/L) | 1.16±0.81 | 1.99±1.35 | ＜0.001 | 1.19±0.82 | 2.07±1.40 | 3.43±3.14 | ＜0.001 |
| HDL cholesterol (mmol/L) | 1.20±0.30 | 1.29±0.41 | 0.057 | 1.20±0.31 | 1.15±0.28 | 1.27±0.21 | 0.780 |
| LDL cholesterol (mmol/L) | 2.59±0.77 | 3.76±0.84 | ＜0.001 | 2.66±0.82 | 3.28±1.18^*^ | 3.11±1.16^*^ | 0.022 |
| FBG (mmol/L) | 5.11±1.10 | 5.68±1.52 | 0.002 | 5.10±1.02 | 7.08±2.72 | 7.08±3.46 | ＜0.001 |
| **Arterial stiffness parameters** |  |  |  |  |  |  |  |
| AVI | 13.54±5.04 | 18.53±6.06 | ＜0.001 | 13.68±5.09 | 22.50±4.58^*^ | 21.50±7.90^*^ | ＜0.001 |

Compared with the low risk group, ^*^ p＜0.05; Compared with the medium risk group, ^#^ p＜0.05

| **Supplementary Table 3. Agreement between two risk** **category in younger than 50 years** | | | | | |
| --- | --- | --- | --- | --- | --- |
| China-PAR risk category | Framingham risk category | | | | |
|  | Low risk | Medium risk | High risk | Kappa | p value |
| Low risk | 518 (91.7%) | 32 (5.7%) | 0 (0.0%) | 0.286 | ＜0.001 |
| Medium risk | 4 (0.7%) | 7 (1.2%) | 0 (0.0%) |  |  |
| High risk | 0 (0.0%) | 4 (0.7%) | 0 (0.0%) |  |  |

| **Agreement between two risk** **category in older than 50 years** | | | | | |
| --- | --- | --- | --- | --- | --- |
| China-PAR risk category | Framingham risk category | | | | |
|  | Low risk | Medium risk | High risk | Kappa | p value |
| Low risk | 91(5.5%) | 313 (18.9%) | 0 (0.0%) | 0.156 | ＜0.001 |
| Medium risk | 5 (0.3%) | 466 (28.2%) | 0 (0.0%) |  |  |
| High risk | 0 (0.0%) | 667(40.3%) | 113 (6.8%) |  |  |

| **Supplementary Table 4 Characteristics of the ≤29 years study population (n=70)** | |
| --- | --- |
| **Traditional risk factors** | n (%) |
| Current smokers | 2 (2.9%) |
| Drinking | 2 (2.9%) |
| Obesity | 5 (7.1%) |
| Diabetes | 5 (7.1%) |
| Hypertension | 4 (5.7%) |
| Dyslipidaemia | 9 (12.9%) |
| **History of medication use** |  |
| Diabetes medications | 0 (0%) |
| Antihypertensive agents | 0 (0%) |
| Lipid-lowering therapy | 0 (0%) |
